# Potential Route of Transmission for Trichodysplasia Spinulosa Polyomavirus

**DOI:** 10.1093/infdis/jix078

**Published:** 2017-02-12

**Authors:** Seweryn Bialasiewicz, Lisa Byrom, Chris Fraser, Julia Clark

**Affiliations:** 1 Child Health Research Centre, The University of Queensland,; 2 Centre for Children’s Health Research, Children’s Health Queensland,; 3 Dermatology Department, Mater Health Services,; 4 School of Medicine, The University of Queensland,; 5 Department of Oncology, Lady Cilento Children’s Hospital, Children’s Health Queensland, and; 6 Infectious Diseases, Immunology/Allergy, Rheumatology, Lady Cilento Children’s Hospital, Children’s Health Queensland, Brisbane, Australia

To the Editor—We read with interest the recent report by van der Meijden et al. [1] in which 2 cases of trichodysplasia spinulosa (TS) in adults were described and data provided to strongly argue for TS being a manifestation of primary virus infection in the context of immunosuppression. The reporting of 2 cases of such a rare disease is of note by itself; however, the hypothesis that primary infection and not reactivation leads to a disease state is of particular interest.

Recently, we encountered a TS case similar to those described by van der Meijden and colleagues [1], with the exception of the patient being a 7- year-old male with pre-B cell acute lymphoblastic leukemia (ALL). Thirty-three months after pre-B-cell ALL diagnosis and initiation of high-risk Children’s Oncology Group chemotherapy study treatment (COG AALL0232) [2], the patient began to develop a facial rash, characterized by generalized flesh-colored folliculocentric papules with fine scale, which worsened with the involvement of the trunk and limbs after the planned cessation of chemotherapy 6 months later. The leukemia remains in remission at the time of last follow-up, and the TS slowly resolved without intervention over a period of 12 months after initial presentation.

Several samples were collected to confirm the clinical diagnosis of TS with the presence of trichodysplasia spinulosa–associated polyomavirus (TSPyV), including saliva and midturbinate flocked swabs (Copan Diagnostics, Italy) resuspended in phosphate-buffered saline; whole blood, from which an aliquot of serum sample was prepared; urine, of which an aliquot was pelleted and resuspended in 1 mL of the original urine; and a punch biopsy of an affected region on the patient’s arm. Sampling occurred 9 months after initial TS presentation, at which point the spicules and cutaneous eruptions had mostly resolved on the face and had moved toward the extremities.

The presence of TSPyV in the punch biopsy was confirmed by a real-time polymerase chain reaction (PCR) targeting the viral protein 1 (VP1) gene [3], with a calculated viral load of 7.7 × 10^3^ genome-equivalents per cell, using endogenous retrovirus 3 as the cellular marker [4]. The virus was also detected in every other collected sample, with the greatest concentration being found within the midturbinate swab (Table 1). To test for encapsidation, aliquots of original nonbiopsy samples were subjected to 2 rounds of recombinant DNase 1 (rDNAse 1; Life Technologies, CA) digestion, followed by routine nucleic acid extraction. Monitoring by real-time PCR [5] of an equine herpesvirus (EHV) DNA spike of known quantity in each sample showed successful digestion of unprotected DNA in all tested samples, apart from whole blood which was inhibited by the presence of ethylenediaminetetraacetic acid (Table 1). Of the samples assayed, TSPyV DNA was protected from digestion only in the midturbinate swab (Table 1), which is highly suggestive of the viral DNA existing in an encapsidated state within the respiratory secretions.

**Table 1.** Quantification of TSPyV Genome Equivalents in Case Patient Samples and Decrease Seen in the Internal rDNAse I control (EHV) and TSPyV Genomes After Sample Digestion Acting As an Encapsidation Assay

		rDNAse 1 Digestion	
Sample	TSPyV, gc/mL^a^	EHV Fold-Decrease	TSPyV Fold-Decrease
Nose swab	1.46E + 07	>200^b^	1
Pelleted urine	1.28E + 05	…	…
Saliva swab	4.10E + 04	206	9
Whole blood	2.20E + 04	1	…^c^
Urine	9.84E + 03	53	ND
Skin biopsy	7.73E + 03	…	…
Serum sample	3.24E + 03	…	…

Abbreviations: EHV, equine herpesvirus; ND, not detected postdigestion; rDNAse 1, recombinant DNase 1; TSPyV, trichodysplasia spinulosa–associated polyomavirus.

^a^Genome copy equivalents per mL of fluid or per cell (biopsy only).

^b^No EHV DNA was detected in digested sample.

^c^Digestion inhibited by ethylenediaminetetraacetic acid.

The detection of TSPyV in multiple body sites is indicative of a disseminated infection, in line with the report of van der Meijden and colleagues [1]. The whole-blood viral load in our patient was substantially lower than that of van der Meijden and colleagues’ 2 patients, which may be reflective of collection at a more advanced time point in the TS symptom progression. Interestingly, the viral load in the whole blood was nearly 10-fold higher than in the serum fraction, which when taken together with van der Meijden and colleagues’ findings of low viral loads in the lymphocyte populations despite high overall viral load in blood, is suggestive of other leukocyte populations being potentially associated with TSPyV. Of particular note, we found that the upper respiratory tract had the highest TSPyV load, the majority of which appeared to be shed as whole virus particles. These findings support the proposal of van der Meijden and colleagues that the upper respiratory tract may be an important site of infection, and support the notion that the respiratory tract may be a potential route of transmission.

## Notes

***Acknowledgments.*** The authors thank the patient and their mother for agreeing to participate in the study.

***Financial support.*** This work was supported by the Children’s Hospital Foundation (grants 50056 and 50006) to S. B.

***Potential conflicts of interest.*** All authors: No reported conflicts. All authors have submitted the ICMJE Form for Disclosure of Potential Conflicts of Interest. Conflicts that the editors consider relevant to the content of the manuscript have been disclosed.
